# Supplementary material for: Genomic Characteristics and Phylogenetic Analyses of a Multiple Drug-Resistant Klebsiella pneumoniae Harboring Plasmid-Mediated MCR-1 Isolated from Tai’an City, China
Source: Pathogens. 2023 Jan 31;12(2):221. doi: 10.3390/pathogens12020221 (PMC9963795; doi:10.3390/pathogens12020221)
Supplement: Supplementary file 1 [file pathogens-12-00221-s001.zip › pathogens-2017190-supplementary.pdf]

Table S1 The accession numberof 100 plasmids

1	KY463454.1	*Escherichia coli* strain WCHEC1618 plasmid pMCR-WCHEC1618, complete sequence
2	KY463451.1	*Escherichia coli* strain WCHEC1606 plasmid pMCR-WCHEC1606, complete sequence
3	MK574665.1	*Escherichia coli* strain DIB-1 plasmid pDIB-1, complete sequence
4	MK836307.1	*Escherichia coli* strain NMG38 plasmid pMCR-NMG38, complete sequence
5	MK172815.1	*Escherichia coli* plasmid pMCR-1-Msc, complete sequence
6	MT929278.1	*Escherichia coli* strain 1253-17-A2 plasmid pMCR-1253-A2, complete sequence
7	CP080094.1	*Salmonella enterica* strain SLR1-8245 plasmid pS8245-3, complete sequence
8	MK869757.1	*Escherichia coli* strain MFDS2258 plasmid pMFDS2258.1
9	MK875285.1	*Escherichia coli* strain MFDS1300 plasmid pMFDS1300.1, complete sequence
10	MK875282.1	*Escherichia coli* strain MFDS1318 plasmid pMFDS1318.1, complete sequence
11	MK869758.1	*Escherichia coli* strain MFDS2254 plasmid pMFDS2254.1
12	CP042970.1	*Escherichia coli* strain CFSAN061769 plasmid pCFSAN061769-01, complete sequence
13	CP018773.2	*Escherichia coli* strain 2016C-3936C1 plasmid pMCR-1-CT, complete sequence
14	MT929280.1	*Klebsiella pneumoniae* strain 1525-17-B1 plasmid pMCR-1525-B1, complete sequence
15	MT929279.1	*Escherichia coli* strain 1449-17-C1 plasmid pMCR-1449-C1, complete sequence
16	MK875286.1	*Escherichia coli* strain MFDS1029 plasmid pMFDS1029.1, complete sequence
17	MF449287.1	*Escherichia coli* strain T71115 plasmid pIBMC-mcr1, complete sequence
18	MT929277.1	*Escherichia coli* strain 1139-17-A1 plasmid pMCR-1139-A1, complete sequence
19	MT929276.1	*Escherichia coli* strain 1138-17-D1 plasmid pMCR-1138-D1, complete sequence
20	MK875284.1	*Escherichia coli* strain MFDS1310 plasmid pMFDS1310.1, complete sequence
21	CP092305.1	*Salmonella enterica* strain PNUSAS070846 plasmid PNUSAS070846-1, complete sequence
22	MT929275.1	*Escherichia coli* strain 1413-17-E1 plasmid pMCR-1413-E1, complete sequence
23	CP092309.1	*Salmonella enterica* strain PNUSAS037276 plasmid PNUSAS037276-2, complete sequence
24	MT929282.1	*Escherichia coli* strain 1525-17-D1 plasmid pMCR-1525-D1, complete sequence
25	CP092316.1	*Salmonella enterica* strain PNUSAS011707 plasmid PNUSAS011707-2, complete sequence
26	CP037906.1	*Escherichia coli* strain LHM10-1 plasmid pLHM10-1-MCR-1, complete sequence
27	CP069685.1	*Escherichia coli* O176:H45 strain MIN9 plasmid pMUB-MIN9-MCR, complete sequence
28	MH938808.1	*Escherichia coli* strain LHM10-1 plasmid pLHM10-1-mcr-1, complete sequence
29	KX254343.1	*Escherichia coli* strain GD-8 plasmid pECGD-8-33, complete sequence
30	KY689634.1	*Escherichia coli* strain 31349 plasmid p31349, complete sequence
31	KY565556.1	*Escherichia coli* strain Mcp0271 plasmid pMcp0271, complete sequence
32	MH143576.1	*Salmonella enterica* subsp. enterica serovar California strain SL01 plasmid pMCR-1, complete sequence
33	MT929289.1	*Escherichia coli* strain 1138-17-A1 plasmid pMCR-1138-A1, complete sequence
34	KX084392.1	*Escherichia coli* strain B65 plasmid pECJS-B65-33, complete sequence
35	LR882921.1	*Escherichia coli* genome assembly, plasmid: 1

36	LR882918.1	*Escherichia coli* genome assembly, plasmid: 1
37	CP063489.1	*Escherichia coli* strain EF7-18-51 plasmid pEF7-18-51-2, complete sequence
38	CP092328.1	*Salmonella enterica* strain PNUSAS020938 plasmid PNUSAS020938-2, complete sequence
39	CP042607.1	*Escherichia coli* strain NCYU-29-19 plasmid pNCYU-29-19-1-MCR1, complete sequence
40	MT929281.1	*Klebsiella pneumoniae* strain 1525-17-C2 plasmid pMCR-1525-C2, complete sequence
41	CP090286.1	*Escherichia coli* strain E-T344-1 plasmid pE-T344-1-mcr-1, complete sequence
42	CP028167.1	*Escherichia coli* strain CFSAN064036 plasmid pGMI17-004-2, complete sequence
43	CP016550.1	*Escherichia coli* strain O177:H21 plasmid unnamed4, complete sequence
44	CP019908.1	*Escherichia coli* strain MDR-56 plasmid pMCR1-NY, complete sequence
45	CP092303.1	*Salmonella enterica* strain PNUSAS047891 plasmid PNUSAS047891-2, complete sequence
46	LC511660.1	*Escherichia coli* 2017.19.01CC plasmid p2017.19.01CC DNA, complete genome
47	CP060967.1	*Escherichia coli* strain EC7 plasmid pEC7-mcr-1, complete sequence
48	CP059291.1	*Escherichia coli* strain 1506 plasmid p1506-3-mcr, complete sequence
49	CP048826.1	*Escherichia coli* strain QEC11-421 plasmid pEcQE11-421-5, complete sequence
50	CP092296.1	*Salmonella enterica* strain PNUSAS070160 plasmid PNUSAS070160-2, complete sequence
51	CP060524.1	*Salmonella enterica* strain SLR1-8250 plasmid pS8250-2, complete sequence
52	CP082794.1	*Klebsiella pneumoniae* strain KP4823 plasmid pKP4823-mcr, complete sequence
53	CP069650.1	*Escherichia coli* O7:H4 strain MIN14 plasmid pMUB-MIN14-MCR, complete sequence
54	LR882927.1	*Escherichia coli* genome assembly, plasmid: 1
55	CP069669.1	*Escherichia coli* O89m:H9 strain MIN11 plasmid pMUB-MIN11-MCR, complete sequence
56	CP069661.1	*Escherichia coli* O89m:H10 strain MIN12 plasmid pMUB-MIN12-MCR, complete sequence
57	CP092330.1	*Salmonella enterica* strain PNUSAS006351 plasmid PNUSAS006351-1, complete sequence
58	MG557853.1	*Escherichia coli* plasmid PN25, complete sequence
59	CP063335.1	*Escherichia coli* strain BS74R-D plasmid pBS74R-D-3, complete sequence
60	CP011290.1	*Salmonella enterica* subsp. diarizonae strain 11-01853 plasmid unnamed1, complete sequence
61	CP060977.1	*Escherichia coli* strain EC4 plasmid pEC4-4, complete sequence
62	CP024919.1	*Klebsiella pneumoniae* strain NH54 plasmid pKPNH54.3, complete sequence
63	CP033849.1	*Escherichia coli* strain FDAARGOS-497 plasmid unnamed3, complete sequence
64	LC479085.1	*Escherichia coli* B2 plasmid pB2 DNA, complete sequence
65	MK477615.1	*Salmonella enterica* strain SH16G1509 plasmid pSH16G1509, complete sequence
66	KX772777.1	*Escherichia coli* strain E15004 plasmid pE15004, complete sequence
67	MW557326.1	*Salmonella enterica* subsp. enterica serovar *Typhimurium* strain 16062 plasmid p16062-MCR.
68	MW010027.1	*Escherichia coli* strain EC1283 plasmid pEC1283, complete sequence
69	MW010026.1	*Escherichia coli* strain EC1280 plasmid pEC1281, complete sequence
70	MW010025.1	*Escherichia coli* strain EC1279 plasmid pEC1279, complete sequence
71	MW010024.1	*Escherichia coli* strain EC1281 plasmid pEC1281, complete sequence

72	LC341397.1	*Escherichia coli* plasmid pEC15-101mcr DNA, complete sequence
73	MN200943.1	*Escherichia coli* strain E648 plasmid pE648MCR-1, complete sequence
74	LC477294.1	*Escherichia coli* A2 plasmid pA2 DNA, complete sequence
75	LC477138.1	*Escherichia coli* A1 plasmid pA1 DNA, complete sequence
76	MK477610.1	*Salmonella enterica* strain SH15G2167 plasmid pSH15G2167, complete sequence
77	MK477604.1	*Salmonella enterica* strain SH15G1397 plasmid pSH15G1397, complete sequence
78	MG257881.1	*Escherichia coli* strain P744T plasmid pP744T-MCR1, complete sequence
79	CP031291.1	*Salmonella enterica* subsp. enterica serovar 4, 12:i:- strain N17-0346 plasmid pN17-0346, complete sequence
80	MG210937.1	*Escherichia coli* strain GZ49260 plasmid pGZ49260, complete sequence
81	AP018411.1	*Klebsiella pneumoniae* plasmid pRYU3223C-1 RYU 3223 DNA, complete sequence
82	MF175191.1	*Escherichia coli* strain CDF8 plasmid pCDF8, complete sequence
83	KX711706.1	*Escherichia coli* strain CSZ4 plasmid pCSZ4, complete sequence
84	MF175185.1	*Escherichia coli* strain PF52 plasmid pPF52, complete sequence
85	MF175184.1	*Escherichia coli* strain PF91 plasmid pPF91, complete sequence
86	KX711707.1	*Escherichia coli* strain FS170G plasmid pFS170G, complete sequence
87	KX129783.1	*Escherichia coli* strain OW3E1 plasmid pOW3E1, complete sequence
88	KX236309.1	*Klebsiella pneumoniae* strain KP-6884 plasmid pMCR1.2-IT, complete sequence
89	MF175186.1	*Escherichia coli* strain PF11 plasmid pPF11, complete sequence
90	KY075660.1	*Escherichia coli* strain GD65 plasmid pGD65-4, complete sequence
91	MT667261.1	*Escherichia coli* strain GDE6P129 plasmid pHNEP129, complete sequence
92	KY075653.1	*Escherichia coli* strain Lishui12 plasmid pLishui12-2, complete sequence
93	KY075652.1	*Escherichia coli* strain GD46 plasmid pGD46-3, complete sequence
94	MF175190.1	*Escherichia coli* strain ColR598 plasmid pColR598-1, complete sequence
95	KY075655.1	*Escherichia coli* strain WH03 plasmid pWH03-3, complete sequence
96	KY471146.1	*Escherichia coli* strain EC111 plasmid pEC111, complete sequence
97	KY120364.1	*Salmonella enterica* subsp. enterica serovar *Typhimurium* strain NG14043 plasmid pNG14043.
98	MW264508.1	*Salmonella enterica* subsp. enterica serovar *Typhimurium* strain S67 plasmid pS67, complete sequence
99	MW264509.1	*Salmonella enterica* subsp. enterica serovar *Typhimurium* strain S69 plasmid pS69, complete sequence
100	MW264507.1	*Salmonella enterica* subsp. enterica serovar *Typhimurium* strain S60 plasmid pS60, complete sequence

Table S2 The virulence gene on chromosome KPTA-2108

VFclass	Virulence factors	Related genes
Adherence	Type 3 fimbriae	*mrkA, mrkB, mrkC, mrkD, mrkF, mrkH, mrkI, mrkJ*
	Type I fimbriae	*fimA, fimB, fimC, fimD, fimE, fimF, fimG, fimH, fimG, fimH, fimI, fimK*
Antiphagocytosis	Capsule	*Capsule I*
Efflux pump	AcrAB	*arcA, acrB*
Iron uptake	Aerobactin	*iucA, iucB, iucC, iucD, iutA*
	Enterobactin	*entA, entB, entC, entD, entE, entF, entS, fepA, fepB, fepC, fepD, fepG, fes*
	Salmochelin	*iroE, iroN*
	Yersiniabactin	*ybtP*
Regulation	RcsAB	*rcsA, rcsB*
	RmpA	*rmpA*
Secretion system	T6SS-I	*clpV/tssH, impA/tssA, ompA, dotU/tssL, hcp/tssD, icmF/tssM, sciN/tssJ, tli1, tle1, tssF, tssG, vasE/tssK, vgrG/tssl, vipA/tssB, vipB/tssC*
	T6SS-II	*clpV*
	T6SS-III	*ompA, dotU, icmF, impA, impF, impG, impH, impJ, lysM, sciN, vgrG*
Serum resistance	LPS rfb locus	*gluP, wbmI, bplA*
Toxin	Colibactin	*clbL, clbG, clbD, clbP*

Table S3 Mobile genetic elements and resistance genes carried by chromosome KPTA-2108

Strain	Length (bp)	Compound transposon (position in contig)	Carrier of resistance factors
chromosome	5306347	cn-9244-IS26 (3836210-3845454)	*rmtB, tet(G),* bla_{TEM-1B}
		cn-2946-IS26 (3844634-3847580)	*bleO*
		cn-18551-IS26 (3818479-3837030)	*qnrB4, sul1,* bla_{DHA-1}, *qacE*
		cn-50151-IS26 (3846761-3896912)	*msr(E), armA, msr(E), mph(E)*

Table S4 Mobile genetic elements and resistance genes carried by plasmid KPTA-2108

Plasmid name	Length (bp)	Plasmid type	Mobile genetic elements (position in contig)	Drug resistance gene (accession number) (position in contig)
pMJ4-1	164378	IncFIB	IS26 (163143-163962)	*bleO* (AF051917) (131623-132016)
			IS26 (4022-4841)	*aac(6')-Ib-cr* (DQ303918) (149103-149702)
			ISKpn24 (39991-42444)	*aadA16* (EU675686) (146922-147767)
			ISEcl1 (43464-44799)	*aph(6)-Id* (M28829) (163971-164378)
			ISKpn26 (49498-50692)	*aac(3)-IId* (EU022314) (160253-161113)
			IS903 (82220-83275)	*aph(3')-Ia* (V00359) (162240-163055)
			IS903 (54251-55307)	*ARR-3* (JF806499) (148554-149006)
			cn-2680-IS26 (161282-163962)	*mph(A)* (D16251) (141337-142242)
			cn-9025-IS903 (54250-83275) (*aph(3')-Ia*)	*sul1* (U12338) (145625-146464)
				sul2 (HQ840942) (1344-2159)
				dfrA27 (FJ459817) (147948-148421)
				tet(A) (AJ517790) (137905-139104)
				aadA16 (EU675686) (146922-147767)
				qacE (X68232) (146524-14680)
				floR (AF118107) (154945-156158)
pMJ4-2	98026	IncI1-I	ISEc37 (11297-13125)	—
pMJ4-3	72739	—	IS26 (47266-48085)	$bla_{CTX\text{-}M\text{-}15}$ (AY044436) (48405-49280)
			ISKpn19 (54291-57139)	$bla_{TEM\text{-}1B}$ (AY458016) (43894-44754)
			cn-11610-ISKpn19 (41025-52635) ($bla_{CTX\text{-}M\text{-}15}$) ($bla_{TEM\text{-}1B}$)	*qnrS1* (AB187515) (52886-53542)
			cn-4640-ISKpn19 (52499-57139) (*qnrS1*)	
pMJ4-44-MCR	30124	IncX4	—	*mcr-1* (KP347127) (25134-26759)
